# Survey on pattern of myopia in school children in Hangzhou after the COVID-19 pandemic: a school-based vision screening study

**DOI:** 10.1186/s12889-024-19338-4

**Published:** 2024-07-11

**Authors:** Ting He, Lei Yin, Qingqing Zheng, Bei He, Zhizi Xu, Tingting Hu, Yuanpeng Wu, Hu Chen, Jie Yu, Ting Shen

**Affiliations:** 1 Eye Center, The Second Affiliated Hospital, School of Medicine, Zhejiang University, Zhejiang Provincial Key Laboratory of Ophthalmology, Zhejiang Provincial Clinical Research Center for Eye Diseases, Zhejiang Provincial Engineering Institute on Eye Diseases, Hangzhou, Zhejiang, CN China; 2 Department of Ophthalmology, Zhejiang Provincial People’s Hospital, Affiliated People’s Hospital, Hangzhou Medical College, Hangzhou, Zhejiang, CN China; 3 Hangzhou Xiaoshan Liuliqiao Hospital, Hangzhou, Zhejiang, CN China

**Keywords:** Myopia, Children, COVID-19, Vision screening, Prevalence

## Abstract

**Background:**

Myopia is a major health issue around the world. Myopia in children has increased significantly during the COVID-19 pandemic in China, but reports are scarce on the prevalence of myopia following the pandemic. This study collected vision screening data of school children in China for five consecutive years to observe the changes in myopia after the pandemic and compare the observed prevalence of myopia before and after the pandemic.

**Methods:**

A school-based vision screening study used stratified samplings to collect the vision screening data in school children aged 6–13 from 45 primary schools in Hangzhou. Vision screening data including uncorrected visual acuity(UCVA) and spherical equivalent refraction(SER). Calculating the mean of SER and the prevalence of myopia and hyperopia from 2019 to 2023.

**Results:**

A total of 79,068 screening results (158,136 eyes) were included in the analysis. A substantial myopic shift (approximately -0.30 diopters [D] on average) was found in 2020 and 2021 compared with 2019 in all age groups and a substantial myopic shift (approximately 0.4 D on average) was found in 2022 compared with 2021. A slight myopic shift (approximately -0.14 D on average) was found in 2023 compared with 2022. The prevalence of myopia in all age groups was the highest for five years in 2020 or 2021, which was 31.3% for 6-year-olds, 43.0% for 7-year-olds, and 53.7% for 8-year-olds. A positive change in the prevalence rate of myopia was found at 6 years old (0.59%, 0.12%, 0.36%, 0.25%, *p* < 0.001). The change in prevalence rate in myopia was shifted slightly in children aged 10–13 years. Children aged 8 to 13 years had a slight increase in myopia prevalence from 2022 to 2023. The prevalence of hyperopia was low and stable in all grade groups, ranging from 0.7% to 2.2% over five years.

**Conclusion:**

Myopia in children has increased rapidly during the COVID-19 pandemic. After the pandemic, the prevalence of myopia in children gradually decreased temporarily and then rebounded. Myopic shift was more apparent in younger children. Myopic shift in children may be related to the reduction of outdoor time, less light, and near work habits, and further research is needed.

## Background

Myopia in children is a major global public health problem, leading to severe visual impairment [1]. Over the past three decades, the prevalence of all levels of myopia has increased rapidly worldwide, especially in East Asia, where the prevalence has reached 80%, creating a significant public health issue [2]. According to the National Health Commission of China, the overall prevalence of myopia in children and adolescents was 52.7%. Among them, 14.3% were 6 years old, 35.6% were elementary students, 71.1% were middle school students, and 80.5% were high school students [3]. Therefore, the myopia of prevention and control is key to the eye health of school children.

Myopia is influenced by both genetic and environmental factors [4], studies have reported that myopia in children currently depends largely on environmental factors and vision-related behavioral activities such as time outdoors, light exposure, near-work-related habits, sleep time, and screen time [5]. The Sunflower Myopia Asian Eye Epidemiology Consortium (AEEC) reported that increased reading and writing and decreased outdoor time were associated with myopia, screen time may be a surrogate factor of near-work or outdoor time, but further research is needed [6]. The protective effect of appropriate outdoor exercise on myopia in children may be related to the unique characteristics of sunlight (intensity, spectral distribution, time pattern, etc.), which cannot be replaced by artificial lighting [7]. The apparent increase in the prevalence of myopia in children and adolescents has a huge impact on public health issues because myopic-related ocular complications can lead to substantial visual loss [8]. Lack of prevention and control of myopia in childhood will increase the risk of high myopia in adulthood [9]. High myopia may increase the risk of myopia-related ocular complications such as subcapsular cataracts, glaucoma, and chorioretinal abnormalities [9, 10]. Therefore, it is important to formulate and implement effective myopia prevention and control strategies in children.

A novel coronavirus epidemic broke out in China in September 2019 and rapidly developed into a worldwide pandemic. To reduce the spread of the pandemic, schools in China were urgently closed in January 2020, and students began home isolation and online teaching [11]. Studies have shown a rapid increase in the prevalence of myopia in students at home isolation, which may be related to the massive use of electronic devices, near work and the reduction in time outdoors [12].

This study described a five-year follow-up period from 2019 to 2023, including the pre-outbreak in December 2019, home isolation from January 2020 to May 2020, return to school around June 2020, irregular home isolation in 2020 and 2021, then study at school normally from 2022 to 2023. Observing the pattern of SER and myopia prevalence in children in Hangzhou provided an important reference for further improving the strategies for preventing and controlling myopia in children.

## Methods

### Study population

Since 2019, vision screenings have been conducted annually for primary school students in Hangzhou, Zhejiang Province, China, from May through June. The time of vision screening was occasionally adjusted because of the pandemic. Our study had 79,068 students (41,477 boys and 37,591 girls) selected from aged 6–13 years in 45 primary schools. There were 31 urban schools and 14 rural schools. The age of children in this study refers to their age on the date of the screening. The study was performed for 5 consecutive years (2019–2023) and all data were analyzed in July 2023. Students screened were from almost the same school, and the increase in individual schools was due to the lifting of home isolation. Vision screening was not performed in some schools in 2020 because of the pandemic. This school-based cross-sectional study was approved by the Ethics Committee of Zhejiang Provincial People's Hospital. All studies were performed following the Declaration of Helsinki and informed consent was obtained from the parents of the participants.

### Vision screening

Parents and children were asked about a history of eye diseases before the screening. Children with amblyopia, low-dose atropine treatment, and significant ocular trauma were excluded from the study. Screening dates were informed to all schools at least two weeks in advance. During the screening process, all children were non-cycloplegic and requested to take off their frame glasses. Optometrists first measured the uncorrected visual acuity (UCVA) using the Bluetooth light visual acuity chart (Snellen chart) and then used the NIDEK Auto Refractometer (NIDEK Ar-1) to obtain the optometric data, including Sphere (S), Cylinder (C), and Axis (A), and take the average value of three measurements. Vision screening included both eyes. All screening procedures were performed by professional optometrists in hospitals and all optometrists and students onwards wore masks during screening [13]. Screening data were recorded from the Teens Optometry Management System. The development of the Teens Optometry Management System in Hangzhou began in January 2019, began to use in June 2019, and the overall function was completed in December 2020. The functional design of the system was referred to the consortium of government bodies in China led by the Ministry of Education released the Comprehensive Plan to Prevent Nearsightedness among Children and Teenagers (CPPNCT) in 2018 [14], and the requirements were proposed by professional ophthalmologists.

### Definitions

The SER was calculated as the S and half of the C. Myopia was defined as SER of − 0.50 D or less. Emmetropia was defined as SER greater than − 0.50 D and less than + 2.00 D. Hyperopia was defined as greater than or equal to + 2.00D. The change in the prevalence rate of myopia was calculated as (prevalence in current year—prevalence in last year) / prevalence in last year × 100(%).

### Statistical analysis

This analysis mainly included spherical equivalent refraction, the prevalence of myopia and hyperopia, and the change in the prevalence rate of myopia. Prevalence rate is the proportion of new and old cases of a disease in the total population at a given time. Spherical equivalent refraction for five years was expressed as mean and standard. One-way analysis of variance was used to compare the annual difference of mean spherical equivalent in different age groups, and LSD multiple comparison tests were used to compare the differences between groups in each year. The prevalence of myopia and hyperopia and the change of prevalence rate in myopia were expressed as frequency (%). The annual prevalence of myopia and hyperopia in different age groups was compared using the Chi-square test. The change in the prevalence rate of myopia between adjacent years was calculated and the difference in the prevalence of myopia between adjacent years was compared by two-proportions Z-test. Analyses were performed using R version 4.1.3, and charts were processed with R studio. Two-sided P values were used and less than 0.05 were considered statistically significant.

## Results

This study had a total of 79,068 screening results (158,136 eyes) and covered the aged 6 to 13 years of children in primary schools, including 41,477(52.5%) boys and 37,591(47.5%) girls. The annual screening is followed by at least 10 of the same schools. The mean of UCVA was 0.21 ± 0.29. The prevalence of myopia among all children in the study was 49.2%, of which 10.7% were moderate myopia and high myopia. The prevalence of hyperopia among all students was 1.3%. Our study found that 267 students had at least one eye with a refractive error less than -8D, and 69 of these students had a refractive error less than -10D.

**Fig. 1:**
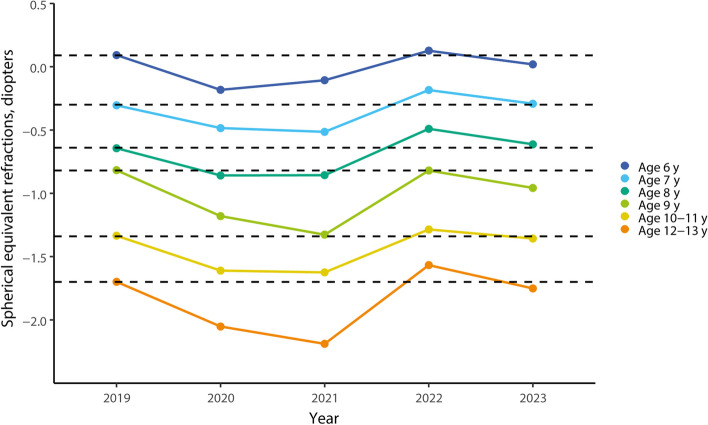
Change in the mean of spherical equivalent refraction (SER) for school children aged 6 to 13 years from 2019 to 2023. The mean of SER in 2019 was used as the baseline and is represented by a dashed line in the figure

The mean of SER in each age group from 2019 to 2023 is shown in Table 1. This study found a substantial myopic shift (approximately -0.30 diopters [D] on average) in 2020 and 2021 compared with 2019 in all age groups. As the pandemic started to slow down, a substantial myopic shift (approximately 0.40 D on average) was found in 2022 compared with 2021. However, the results showed a slight myopic shift (approximately -0.14 D) in 2023 compared with 2022, and the mean of SER in 2023 was similar to that in 2019 in all age groups. In Fig. 1 shows a substantial myopic shift in 2020 and 2021 due to the impact of the pandemic. The mean of SER in 2019 as a baseline, the mean of SER almost returned to the baseline from 2022 after the pandemic.

**Table 1:** The mean of spherical equivalent refraction (SER) for each year in school children

**Age, y**	**n**	**SER, mean(SEM)**					
		**2019**	**2020**	**2021**	**2022**	**2023**	***P*** value^*^
6	13,752	0.09(0.93)	-0.18(1.14)^a^	-0.10(1.11)^a^	0.12(0.98)	0.02(0.78)	< 0.001
7	13,556	-0.30(1.18)	-0.49(1.34)^a^	-0.51(1.27)^a^	-0.19(1.14)^a^	-0.29(1.01)	< 0.001
8	13,192	-0.64(1.28)	-0.86(1.45)^a^	-0.86(1.47)^a^	-0.49(1.31)^a^	-0.62(1.26)	< 0.001
9	14,172	-0.82(1.35)	-1.18(1.54)^a^	-1.33(1.62)^a^	-0.82(1.59)	-1.00(1.50)	< 0.001
10–11	12,547	-1.34(1.67)	-1.61(1.86)^a^	-1.63(1.82)^a^	-1.29(1.72)	-1.36(1.63)	< 0.001
12–13	11,866	-1.70(1.78)	-2.05(1.98)^a^	-2.19(2.01)^a^	-1.54(1.88)	-1.77(1.85)	< 0.001

*Abbreviation: SER* Spherical equivalent refraction, *SEM* The mean of spherical equivalent refraction

^*^Comparing the variance for SERs across 5 years within each age group using one-way analysis of variance

^a^ Significant difference when compared with 2019 (*p* < 0.01)

**Fig. 2:**
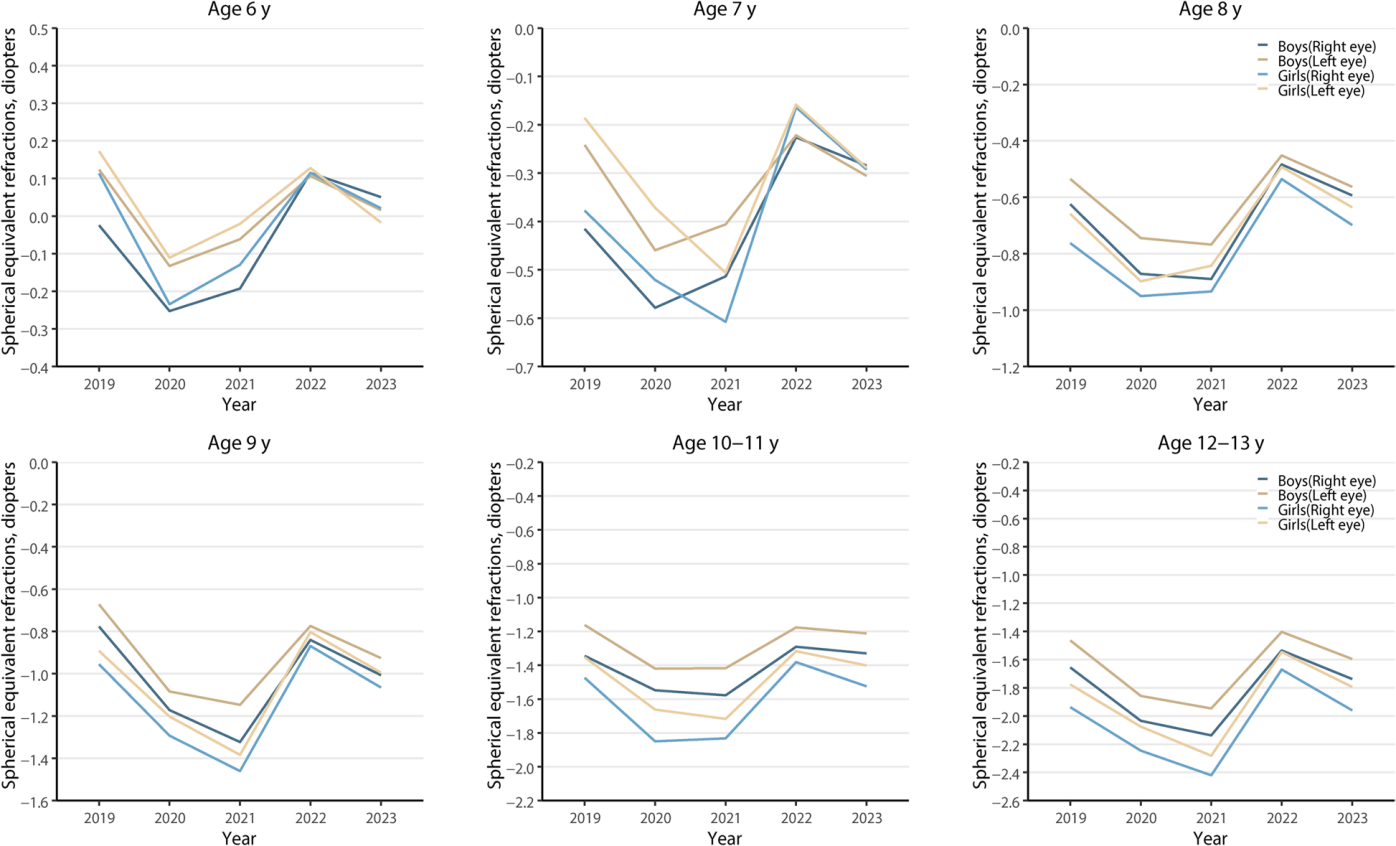
Changes in the mean of spherical equivalent refraction (SER) in different groups of children aged 6 to 13 years from 2019 to 2023

Figure 2 further showed that the SER was divided into the right eye group of boys, the left eye group of boys, the right eye group of girls, and the left eye group of girls to observe the myopic shift of left and right eyes in boys and girls. It found that the right eyes were more myopic than the left in all children and girls were more likely to become myopic than boys with age.

The prevalence of myopia and hyperopia in school children from 2019 to 2023 is shown in Table 2. During the five-year follow-up, the prevalence of myopia in all age groups peaked in 2020 or 2021. The prevalence of myopia peaked in children aged 6 and 8 years in 2020, with a prevalence as high as 31.0% and 53.7%, and other age groups all peaked in 2021, with a prevalence of 43.0%, 67.8%, 72.8% and 81.9%, respectively. In 2022, the prevalence of myopia decreased in all age groups. In 2023, we found a slight decrease in the prevalence of myopia in children aged 6 and 7 years, but a slight increase in the prevalence of myopia in children aged 8 to 13 years. The change in the prevalence of myopia in children aged 6 to 13 years was significant over the five years(*p* < 0.001). The prevalence of hyperopia in all children was low and the changes were relatively stable.

**Table 2:** Prevalence of myopia and hyperopia for each year in school children (Hangzhou, China, 2023)

**Age, y**	**n**	**Prevalence per year(%)**										***P*** value^*^		
		**2019**		**2020**		**2021**		**2022**		**2023**				
		M	H	M	H	M	H	M	H	M	H	M		H
6	13,752	19.7	1.4	31.3^#^	1.2	27.6	2.2	17.7	1.9	13.2	1.1	< 0.001		< 0.001
7	13,556	31.9	0.9	42.1	1.8	43.0^#^	1.5	29.1	1.8	27.8	1.1	< 0.001		< 0.001
8	13,192	47.6	1.7	53.7^#^	1.3	53.5	1.3	40.2	1.4	41.0	1.0	< 0.001		0.103
9	14,172	52.7	1.7	64.1	1.1	67.8^#^	1.2	51.4	1.6	52.2	1.1	< 0.001		0.033
10–11	12,547	66.7	0.9	71.8	1.3	72.8^#^	1.3	62.1	1.0	63.1	0.7	< 0.001		0.004
12–13	11,866	72.5	0.8	77.1	1.0	81.9^#^	1.2	66.5	1.1	70.4	0.8	< 0.001		0.063

*Abbreviation: M* Myopia, *H* Hyperopia

Myopia was defined as SER of − 0.50 D or less. Hyperopia was defined as SER of + 2.00 D or greater

^#^The highest myopia prevalence was from 2019 to 2023. *Comparing the variance for the prevalence of myopia and hyperopia across 5 years within each age group using the Chi-square test

**Fig. 3:**
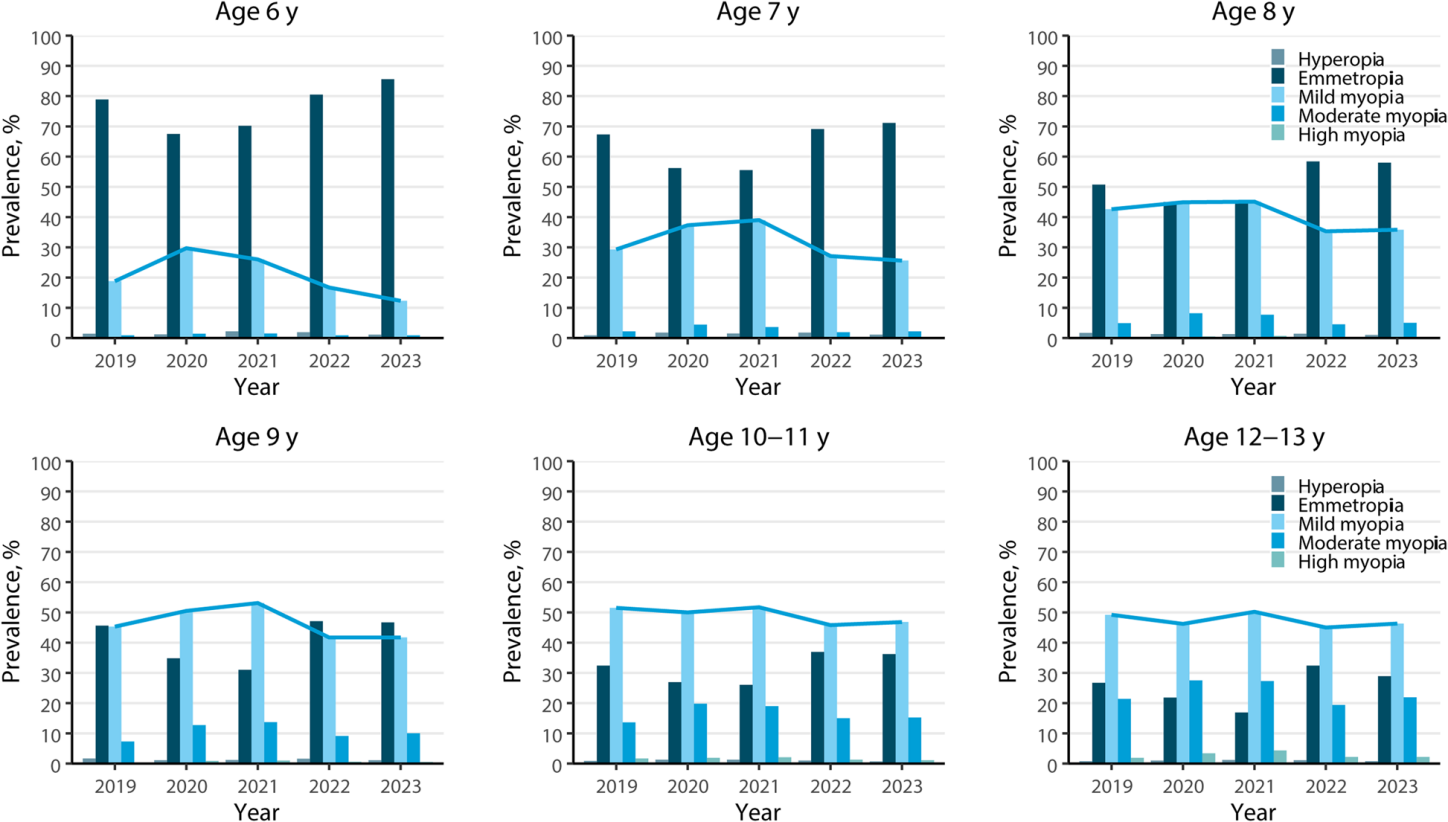
Prevalence of myopia and hyperopia for school children aged 6–13 in 2019–2023. Mild myopia: -3.00 D < SER ⩽ -0.50 D; Moderate myopia: -6.00D ⩽ SER ⩽-3.00 D; High myopia: SER < -6.00D; Emmetropia: -0.50 D ⩽ SER < +2.00 D; Hyperopia: SER ≥ +2.00 D. The blue lines represent the trends in mild myopia prevalence

After further grouping myopia and hyperopia as shown in Fig. 3, the results showed that the number of students with high myopia increased and low hyperopia decreased with age. The prevalence of mild myopia in all age groups decreased significantly in 2022, but a slightly increasing trend was observed in myopia prevalence in children aged 8 to 13 years from 2022 to 2023.

The change in the prevalence rate of myopia in children is shown in Table 3. The rate of myopic shift in children aged 6 was the most significant when compared to other age groups. The prevalence of myopia in children aged 10–13 years shifted slowly. In the five-year prevalence of myopia in children, there is a positive growth rate from 2019 to 2020 and from 2020 to 2021(except for the six-year-old group). A negative growth rate from 2021 to 2022 was shown in all age groups. The prevalence of myopia in children aged 6 and 7 years showed a negative growth from 2022 to 2023. However, changes in the prevalence of myopia in children aged 8 to 13 years showed a positive growth but not significant.

**Table 3:** Change in the prevalence of myopia for each year in school children in 2019–2023

Age, y	n	Change in Prevalence (%), myopia							
		**2019- 2020**		**2020—2021**		**2021—2022**		**2022—2023**	
		**Δ PR**	***P*** value^*^	**Δ PR**	***P*** value^*^	**Δ PR**	***P*** value^*^	**Δ PR**	*P* value^*^
6	13,752	0.59	0.001	-0.12	0.001	-0.36	0.001	-0.25	0.001
7	13,556	0.32	0.001	0.02	0.394	-0.32	0.001	-0.04	0.076
8	13,192	0.13	0.001	0.00	0.876	-0.25	0.001	0.02	0.346
9	14,172	0.22	0.001	0.06	0.001	-0.24	0.001	0.02	0.275
10–11	12,547	0.08	0.002	0.01	0.335	-0.15	0.001	0.02	0.217
12–13	11,866	0.06	0.004	0.06	0.001	-0.19	0.001	0.06	0.001

*Abbreviation*: *PR* Prevalence rate. Δ PR, change in prevalence rate

^*^Comparing the prevalence rate in myopia within each age group using the two-proportions z test

## Discussion

Vision screening in students was conducted through the release of the *Comprehensive Plan to Prevent Nearsightedness among Children and Teenagers* (CPPNCT) in 2019, it aims to monitor and prevent myopia in children and adolescents and achieve early detection and treatment. Myopia in children has shifted significantly during the COVID-19 epidemic, then it decreased after the pandemic slowed down. However, we found that the prevalence of myopia in children aged 8 to 13 years in 2023 almost rebounded to the pre-pandemic situation. We found that the prevalence of myopia in children aged 6 and 7 years decreased from 2022 to 2023, but the myopic shift in younger children was more unstable and they were still at the risk of developing myopia. This reminds us of the importance of continuing to monitor changes in myopia in children and adolescents, and to strengthen and refine the management of myopia prevention and control among children and adolescents.

A consortium of government bodies in China adopted the management of closing schools and home isolation from January to May 2020 to reduce the spread of COVID-19 among children. With home isolation reduced time outdoors and online teaching increased the use of electronic devices and near-work time [12, 15], our study found a significant increase in myopic prevalence in all age groups in 2020 and 2021 with an outbreak of the pandemic, which is consistent with the results of Hu et al. [11]. Time outdoors is a crucial factor against the onset of myopia [16]. One study with samples from 51 Sydney schools showed higher levels of total time spent outdoors were associated with less myopia and more hyperopia mean refraction after adjusting for near work, parental myopia, and ethnicity [17]. The protective effect of outdoor time may be related to exposure time and light intensity [18]. Bright light can stimulate the retina to release dopamine, inhibit the increase of axial length, and control myopia to a certain extent, the mechanism has been confirmed in animal experiments [19]. Outdoor light is more protective against myopia than indoor light may be related to differences in the composition of the spectrum [4, 20]. Reduced outdoor exercise and the heavy use of electronic devices have increased near-work time and near-work-related habits, more time spent on near-work activities was associated with higher odds of myopia [21]. Our study found that myopia prevalence in children begins to decrease in 2022 and the myopia prevalence in 2022 was the lowest in the five-year follow-up, benefiting from the implementation of myopia prevention and control policies. Unexpectedly, the prevalence of myopia appeared to increase slightly in 2023. Our study found that the post-pandemic decrease in the prevalence of myopia in children aged 8 to 13 years was followed by an increase, which may be related to an increase in homework and the habits and time of near-work after returning to school, but further investigation is needed [3]. High educational loads were associated with the high prevalence of myopia [3]. Reducing the burden of studying is an important way to prevent and control myopia in children. These efforts need to be made in conjunction with parents, schools, and the community. Continuous improvement and enhancement of myopia prevention and control measures are important for the long-term prevention of myopia.

This study found the prevalence of hyperopia was low and stable in all grade groups, ranging from 0.7% to 2.2% over five years. The development of hyperopia in children follows a natural progression and does not affect visual development within the normal range [22]. If children have hyperopia out of range, it may cause amblyopia [23]. A certain proportion of children with amblyopia are not timely prevented due to moderate and high hyperopia [24, 25]. The refractive development of children is a process from hyperopia to emmetropia, and the continuous development after emmetropia forms myopia [22]. It is important to pay attention to the hyperopia state before emmetropia, called hyperopia reserve, which may be used as a key indicator for early prediction of myopia [26]. When the hyperopic reserve is insufficient, it increases the incidence of myopia [22]. A cohort study demonstrated that a greater hyperopia reserve at baseline is associated with a reduced likelihood of developing myopia [27]. It is crucial to monitor the hyperopic reserve in younger children. Parents should regularly take their children to an ophthalmic hospital every 3 to 6 months to check for refractive errors, regardless of the age of the children and whether they have myopia or hyperopia.

In this study, myopia rose with age in children, which may conform to the pattern of myopia progression because axial length growth is irreversible [28]. Axial length is one of the main factors determining refractive error in children [29]. With the increase in axial length, the degree of myopia will be higher. The axial length in children changes gradually with growth and development [30]. School children are in a critical period of growth and development, and the increase of myopia in children is closely related to the growth of axial length [31]. It is important to focus on the development of axial length in school children and to intervene in time to prevent myopia once they have exceeded the standard length of the axial length for that age group.

Our study showed a remarkable shift in myopia in children aged 6–8 years and a slight change in myopia in children aged 9–11 years. A medical big data used in this multicenter study in China demonstrated that the age of myopia onset was 7.47 ± 1.67 years and that myopia progresses rapidly between the ages of 5 and 11 years, and then largely stabilizes [32]. This may be related to the development of behavioral habits that lead to myopia [33], as younger children lack consciousness to prevent myopia. Parents should timely pay close attention to the near-work habits of young children and increase the outdoor exercise time with their children. Our study also found the prevalence rate of myopia in children aged 6 years was more than 10%, with the highest reaching 31.6%. Focusing on the development of myopia in younger children is crucial, young children are the best stage to prevent and control myopia [34, 35]. For example, orthokeratology has a significant effect on the control of myopia in young children [36]. These findings highlight the need to strengthen the prevention and control of myopia in younger children.

This study observed that girls were more likely to be myopic than boys, which is generally consistent with previous studies [12, 15]. In an epidemiological study, female gender was found to be an independent risk factor for myopia (odds ratio, 1.24; 95% CI, 1.21–1.27) [34]. A relatively novel idea is that sex differences in the prevalence of myopia may be related to different ages of pubertal development and estrogen level changes [12, 37]. Girls enter puberty earlier than boys [38], which may lead to a higher rate of axial length growth in girls than in boys, resulting in a higher prevalence of myopia in girls than boys in school children. Gong et al. found that the myopia degree increased along with the decrease in estrogen level, the relationship between estrogen and myopia in children is rarely reported. Estrogen affects the content of matrix metalloproteinase‑2 (MMP‑2) and/or MMP‑9 in the sclera of human ocular cells and the scleral remodeling through MMP‑2 upregulation [37]. The reason and mechanism are not fully understood and need to be further studied. With the basic research, a new platform can be built for the clinical application of estrogen and myopia in children. Some studies think that sex differences in myopia may be related to behavioral habits, such as boys being more willing to spend time outdoors than girls, but this remains speculative [33]. Based on the above discussion, our study thought customization and personalization in myopia prevention and control strategies can be adopted for boys and girls. In addition, Our study found that emmetropization of the right eye was more likely to be affected than that of the left eye, and became the future development of myopia. A population-based screening study reported differences in SER in the left and right eyes, they think this may be related to the development of anisometropia in school children [12]. The differences in the left and right eyes may be related to the dominant eye [39]. One study showed that the dominant eye may have an effect on emmetropization than the non-dominant eye and that the right eye was the dominant eye in 76% of subjects [40]. The mechanism of the difference between the left and right eyes needs to be further explored.

Effective strategies to control the prevalence of myopia in school children are essential, with particular reference to vision screening [41]. Large-scale vision screening provides a scientific basis for the effectiveness of myopia prevention and control strategies [42]. School vision screening made students with abnormal visual acuity get the attention and care of teachers. Parents can receive a report on the visual acuity of their children and be prompted to take their children to a professional eye examination in hospitals to achieve early detection and treatment. Parents are paying more attention to the prevention and control of myopia in children. Those proved the effectiveness of vision screening. In addition, Axial length, which was not measured in our study, is an important predictor of myopia [43, 44]. It is worth noting that the inclusion of ocular biological parameters such as axial length and corneal curvature in vision screening programs could further analyze the underlying biological processes of myopia. A refined vision screening can establish a complete vision screening system for children, which is important to improve the prevention and control strategy of myopia in children.

This study is a large school-based vision screening study and has over a long period of five-year follow-up. The schools screened included both rural and urban areas, which reduced regional bias. This study also has some limitations. firstly, Our study did not use measurement data after cycloplegia, which may cause measurement bias. Because of the large number of schools and limited resources, cycloplegic refraction was difficult to apply to all students. Secondly, the population tested varied from year to year because of the pandemic, and the presence of graduates and new students each year, so these differences were presented as population averages. Thirdly, there were many missing values in the biological parameters such as axial length and corneal curvature measured by screening, so these biological parameters were not used in this study. In the future, our research will keep following up with more comprehensive vision screening over the long term, combined with ocular biometry parameters.

## Conclusion

Our study found myopia in children increased rapidly during the COVID-19 pandemic from 2019 to 2021. The prevalence of myopia in children aged 6 to 13 years peaked at 31.3%, 43.0%, 53.7%, 67.8%, 72.8%, and 81.9% in 2020 or 2021. After the pandemic, the prevalence of myopia in children gradually decreased and returned to baseline in 2022. The prevalence of myopia among children ages 6 to 13 was 17.7%, 29.1%, 40.2%, 51.4%, 62.1% and 66.5% in 2022, respectively. However, a slight increase (approximately + 0.9% diopters [D] on average) in myopia prevalence was found in children aged 8–13 years in 2023. Myopia in younger children shifted significantly, and the prevention and control measures were more effective in younger children, which reminded us to pay more attention to this population. We should keep constant concern about the prevention and control of myopia in post-pandemic times. Refining vision screening items, in terms of adding axial length and corneal curvature, establishing a complete vision screening system for children, and keeping track, may lead to a higher level of myopia control.

## Data Availability

All data generated or analyzed during this study are included in this published article.

## Abbreviations

UCVA: Uncorrected visual acuity
SER: Spherical equivalent refraction
D: Diopters
DS: Sphere diopter
DC: Cylinder diopter
CPPNCT: Comprehensive Plan to Prevent Nearsightedness among Children and Teenagers
