# Correction: Survey on pattern of myopia in school children in Hangzhou after the COVID-19 pandemic: a school-based vision screening study

**DOI:** 10.1186/s12889-024-19801-2

**Published:** 2024-08-29

**Authors:** Ting He, Lei Yin, Qingqing Zheng, Bei He, Zhizi Xu, Tingting Hu, Yuanpeng Wu, Hu Chen, Jie Yu, Ting Shen

**Affiliations:** 1 Eye Center, The Second Affiliated Hospital, School of Medicine, Zhejiang Provincial Key Laboratory of Ophthalmology, Zhejiang Provincial Clinical Research Center for Eye Diseases, Zhejiang University, Zhejiang Provincial Engineering Institute on Eye Diseases, Hangzhou, Zhejiang, CN China; 2 Department of Ophthalmology, Affiliated People’s Hospital, Zhejiang Provincial People’s Hospital, Hangzhou Medical College, Hangzhou, Zhejiang, CN China; 3 Hangzhou Xiaoshan Liuliqiao Hospital, Hangzhou, Zhejiang, CN China

**BMC Public Health (2024) 24:1850**

10.1186/s12889-024-19338-4

The original publication of this article contained an incorrect caption for Fig. 3. The incorrect and correct caption are shown in this correction article, the original article has been updated.

Incorrect

Prevalence of myopia and hyperopia for school children aged 6–13 in 2019–2023. Mild myopia: -3.00 D < SER ⩽ -0.50 D; Moderate myopia: -6.00D ⩽ SER ⩽ -3.00 D; High myopia: SER < -6.00D; Emmetropia: -0.50 D ⩽ SER < + 2.00 D; Hyperopia: SER ≥ +2.00 D. The blue lines represent the trends in mild myopia prevalence.

Correct

Prevalence of myopia and hyperopia for school children aged 6–13 in 2019–2023. Mild myopia: -3.00 D < SER ⩽ -0.50 D; Moderate myopia: -6.00D ⩽ SER ⩽ -3.00 D; High myopia: SER < -6.00D; Emmetropia: -0.50 D ⩽ SER < + 2.00 D; Hyperopia: SER ≥ + 2.00 D. The blue lines represent the trends in mild myopia prevalence.

